# Androgen receptor signaling in the lungs mitigates inflammation and improves the outcome of influenza in mice

**DOI:** 10.1371/journal.ppat.1008506

**Published:** 2020-07-09

**Authors:** Landon G. vom Steeg, Santosh Dhakal, Yishak A. Woldetsadik, Han-Sol Park, Kathleen R. Mulka, Emma C. Reilly, David J. Topham, Sabra L. Klein

**Affiliations:** 1 W. Harry Feinstone Department of Molecular Microbiology and Immunology, The Johns Hopkins Bloomberg School of Public Health, Baltimore, Maryland, United States of America; 2 Department of Molecular and Comparative Pathobiology, The Johns Hopkins School of Medicine, Baltimore, Maryland, United States of America; 3 Department of Microbiology and Immunology, University of Rochester Medical Center, School of Medicine and Dentistry, Rochester, New York, United States of America; 4 Department of Biochemistry and Molecular Biology, The Johns Hopkins Bloomberg School of Public Health, Baltimore, Maryland, United States of America; St. Jude Children's Research Hospital, UNITED STATES

## Abstract

Circulating androgens can modulate immune cell activity, but the impact of androgens on viral pathogenesis remains unclear. Previous data demonstrate that testosterone reduces the severity of influenza A virus (IAV) infection in male mice by mitigating pulmonary inflammation rather than by affecting viral replication. To examine the immune responses mediated by testosterone to mitigate IAV-induced inflammation, adult male mice remained gonadally intact or were gonadectomized and treated with either placebo or androgen-filled (i.e., testosterone or dihydrotestosterone) capsules prior to sublethal IAV infection. Like intact males, treatment of gonadectomized males with androgens improved the outcome of IAV infection, which was not mediated by changes in the control of virus replication or pulmonary cytokine activity. Instead, androgens accelerated pulmonary leukocyte contraction to limit inflammation. To identify which immune cells were contracting in response to androgens, the composition of pulmonary cellular infiltrates was analyzed and revealed that androgens specifically accelerated the contraction of total pulmonary inflammatory monocytes during peak disease, as well as CD8^+^ T cells, IAV-specific CD8^+^ T numbers, cytokine production and degranulation by IAV-specific CD8^+^ T cells, and the influx of eosinophils into the lungs following clearance of IAV. Neither depletion of eosinophils nor adoptive transfer of CD8^+^ T cells could reverse the ability of testosterone to protect males against IAV suggesting these were secondary immunologic effects. The effects of testosterone on the contraction of immune cell numbers and activity were blocked by co-administration of the androgen receptor antagonist flutamide and mimicked by treatment with dihydrotestosterone, which was also able to reduce the severity of IAV in female mice. These data suggest that androgen receptor signaling creates a local pulmonary environment that promotes downregulation of detrimental inflammatory immune responses to protect against prolonged influenza disease.

## Author summary

In the United States alone, it is estimated that over 2 million men are taking testosterone replacement therapy caused by congenital, acquired, or age-associated reductions in circulating testosterone, with known immunomodulatory effects. Despite the increasing popularity of testosterone replacement therapy, the influence of testosterone deficiency and treatment on clinical outcomes of infectious disease has not been adequately considered. Disease following influenza A virus (IAV) infection is largely immune-mediated, with severe disease often associated with excessive or aberrant immune responses (i.e., a cytokine storm) to the virus. We have made the novel observation that administration of testosterone to male mice improves the outcome of IAV infection not by mitigating global pulmonary cytokine production, but by promoting the specific contraction of pulmonary inflammatory monocytes during peak disease and the frequencies of virus-specific pulmonary CD8^+^ T cells and eosinophils in the lungs following control of viral replication. The protective effects of testosterone on IAV pathogenesis are dependent on androgen receptor signaling, which creates a pulmonary environment conducive to reduced pulmonary inflammation. Rather than acting directly on a single cell population, androgen receptor signaling has multicellular effects and creates a local environment that promotes accelerated contraction of inflammatory immune cells. Activation of androgen receptor signaling confers protection during IAV infection by modulating the immune response, which may have therapeutic potential in both male and female patients.

## Introduction

Testosterone is a sex steroid hormone produced and released primarily by Leydig cells in the testes of males, which has significant effects on health and disease [1]. In men, low testosterone, whether congenital, acquired, or age-related, is associated with an increased risk of all-cause and cardiovascular-related mortality [2–4]. Additionally, low testosterone in males has been linked to metabolic dysfunction, osteoporosis, muscle weakness, fatigue, cognitive impairment, and sexual dysfunction; while in hypogonadal men, testosterone replacement therapy has been shown to improve cardiovascular disease outcomes, increase quality of life perceptions, and improve age-associated anemia [4–9]. Although safety concerns exist, the perceived benefits of testosterone replacement therapies have resulted in a dramatic increase in its therapeutic use over the last two decades, with an estimated 2.3 million men undergoing testosterone replacement therapy in the United States alone in 2013 [10, 11]. Included in these numbers is a 4-fold increase in testosterone replacement therapy use in reproductively aged males (i.e. 18 to 45 years of age), a demographic often overlooked in studies of the implications of low testosterone [12]. Despite the increasing popularity of testosterone replacement therapy, the influence of testosterone deficiency and treatment on clinical outcomes of infectious disease has not been adequately considered.

The biological effects of testosterone are typically mediated through androgen receptor (AR) signaling [2, 13]. Intracellular ARs are present in cells throughout the body, with testosterone modulating the activities of a variety of tissue and cell types [2]. Notably, ARs are widely expressed in cells of both the innate and adaptive immune system, including macrophages, neutrophils, and T cells [2, 13]. In humans and nonhuman animals, testosterone and its physiologically active metabolite, dihydrotestosterone (DHT), are broadly immunomodulatory and capable of altering the number, function, and differentiation of most immune cell populations. For example, in the presence of testosterone, murine macrophages increase IL-10 and decrease TNFα synthesis, while T cell numbers and activity (e.g., IL-4 and Il-12 production) are reduced [14, 15]. In adult human males, clinical depletion of testosterone decreases regulatory T cell numbers (Tregs), reduces mitogen-induced IFNγ expression in CD8^+^ T cells, and suppresses the ability of natural killer cells to proliferate [16]. In contrast, production of cytokines, including TNFα and IL-1β, by monocytes, is enhanced in human males compared with females suggesting a possible immunostimulatory role for testosterone in some cell populations [17] and that sex differences and the immunomodulatory effects of sex steroids can vary based on cell type [18]. Although, the immunomodulatory properties of testosterone are established, the impact of low testosterone on the severity of viral infection remains incompletely characterized. If testosterone is capable of broadly regulating the immune system, then in viral infections where pathogenesis is driven by the immune response rather than viral replication, testosterone is likely to reduce the severity of infection.

Disease following influenza A virus (IAV) infection is largely immune-mediated, with severe disease often associated with excessive or aberrant immune responses (i.e., a cytokine storm) to the virus [19, 20]. We have previously shown that low testosterone in males, whether age-related or surgically-induced, increases the severity of IAV infection [21–23]. Furthermore, these changes are associated with delayed resolution of pulmonary inflammation (i.e., following control of viral replication), independent of either changes in viral replication or induction of growth factors (e.g., amphiregulin), suggesting that the protective effects of testosterone are mediated through downregulation of the inflammatory response during infection [22, 23]. In the current study, we sought to characterize the effects of testosterone on the immune response to IAV using a murine model of IAV infection. We show that testosterone improves the outcomes of IAV infection not by mitigating the cytokine storm, but by promoting the contraction of pulmonary inflammatory monocytes during peak disease and virus-specific pulmonary CD8^+^ T cells and eosinophils in the lungs following control of viral replication. Neither depletion of eosinophils nor adoptive transfer of CD8^+^ T cells could mitigate the protective effects of androgens on IAV pathogenesis, suggesting these were secondary effects that were a consequence and not the cause of androgen receptor signaling-mediated protection. The protective effects of androgen receptor signaling in the lungs during IAV pathogenesis were present in both males and females and may have broad therapeutic potential.

## Results

### Testosterone reduces the severity of IAV infection in male mice

**Fig 1:**
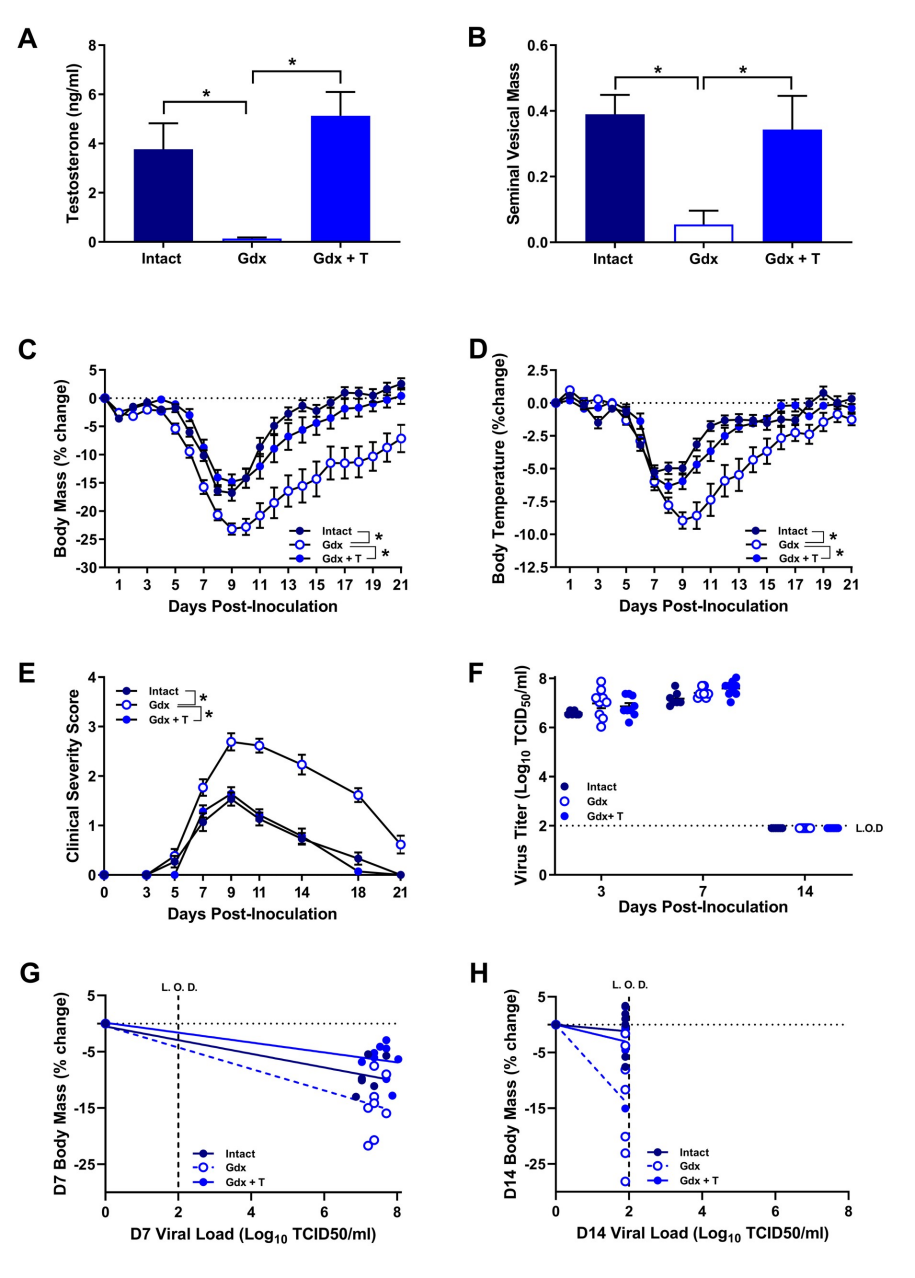
Testosterone depletion increases the severity of influenza A virus (IAV) infection. Adult male mice were gonadectomized and implanted with either testosterone (gdx + T) or placebo (gdx) containing capsules or received sham surgeries (intact) prior to inoculation with a sub-lethal dose of ma2009 H1N1 IAV. Plasma and seminal vesicles were collected at 21 days post inoculation (dpi) and testosterone concentrations (A) were analyzed by ELISA, while seminal vesicle mass as a percentage of total body mass was calculated as a biomarker of androgenic activity (B; n = 11-12/treatment). Mice (n = 13-15/treatment) were monitored daily for changes in body mass (C), body temperature (D), and clinical disease severity (E). Infectious virus was measured in the lungs by TCID_50_ at 3, 7, and 14 dpi (F; n = 6-9/treatment/time-point). The correlation between body mass and virus titer at 7 and 14 dpi, was quantified as a measure of disease tolerance using linear regression (G and H; n = 6-9/treatment/time-point). Data represent means +/- SEM from two independent replications and significant differences between treatment groups are denoted by asterisks (*p < 0.05).

To reproduce and confirm previous reported effects of testosterone on the severity of IAV infection [21, 22], adult male mice underwent sham surgeries or were gonadectomized and received either testosterone or placebo capsules. Gonadectomized males had significantly lower concentrations of circulating testosterone and seminal vesicle mass (i.e., androgen responsive tissue that can be used as a biomarker of circulating concentrations) than either gonad-intact or gonadectomized males that received testosterone (**Fig 1A and 1B**; *p* < 0.05). Following intranasal inoculation with a sub-lethal dose of mouse-adapted (ma)2009 H1N1, mice were monitored for 21 days post inoculation (dpi) for changes in body mass, body temperature, and clinical disease severity. Similar to previous studies [21, 22], testosterone-depleted mice experienced greater body mass loss, hypothermia, and clinical disease severity than either gonad-intact or gonadectomized males that received testosterone (**Fig 1C–1E**; *p* < 0.05). Despite these differences in the severity of IAV infection between testosterone-depleted and testosterone-replete mice, neither peak virus titers (i.e., the highest measured) at 7 dpi nor the clearance of detectable infectious virus from the lungs by 14 dpi was affected by testosterone concentrations in males (**Fig 1F**), suggesting that testosterone does not alter resistance to IAV infection. An alternate strategy that can be employed by hosts to mitigate the detrimental effects of infection is disease tolerance, which reduces the fitness costs of infection independent of changes in pathogen survival [24, 25]. To test whether testosterone made males more tolerant to IAV infection, we analyzed the interaction between hypothermia and pulmonary viral load at 7 and 14 dpi. Testosterone-replete males experienced less body mass loss relative to viral load than placebo-treated males during both peak viral titers (7 dpi) and following control of viral replication (14 dpi; **Fig 1G and 1H**; *p* < 0.05 in each case). These data suggest that testosterone reduces the severity of IAV infection by making males more tolerant, rather resistant, to IAV infection.

### Pulmonary cytokine and chemokine concentrations are not altered by testosterone in males

**Fig 2:**
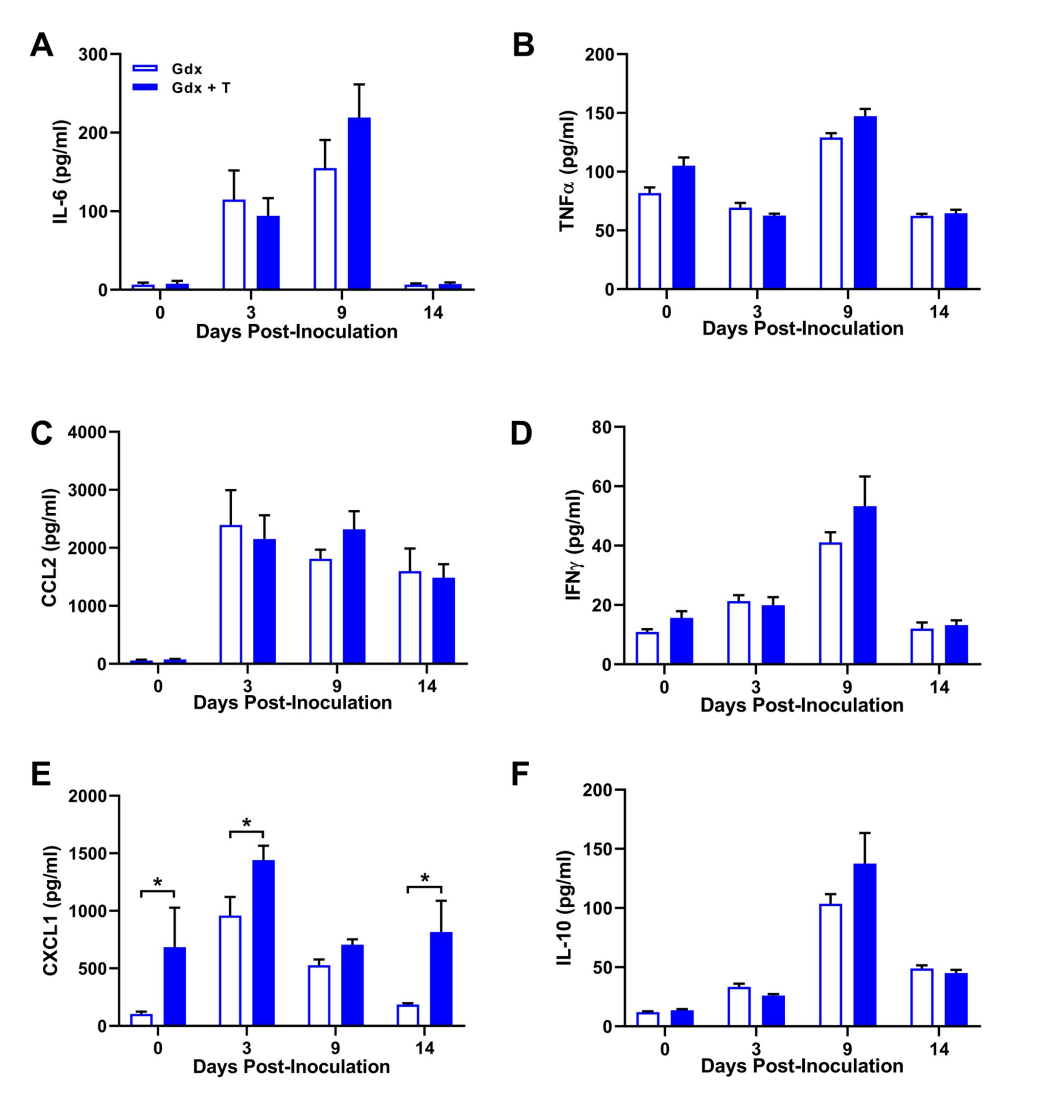
Testosterone does not alter pulmonary cytokine or chemokine concentration during influenza A virus (IAV) infection. Adult male mice were gonadectomized and implanted with either testosterone (gdx + T) or placebo (gdx) containing capsules, and then inoculated with a sub-lethal dose of ma2009 H1N1 IAV or were mock infected. At 0, 3, 9, or 14 dpi (n = 8-11/treatment/time-point), lung tissue was collected and homogenized, and cell free supernatants were used to quantify pulmonary concentrations of IL-6 (A), TNFα (B), CCL2 (C), IFNγ (D), CXCL1 (E), and IL-10 (F). Data represent means +/- SEM from two independent replications and significant differences between treatment groups are denoted by asterisks (*p < 0.05).

The severity of IAV infection is associated with induction of pulmonary cytokine and chemokine responses, which can lead to excessive cellular infiltration, pulmonary inflammation, and tissue damage, if improperly regulated [26, 27]. To test whether testosterone altered the kinetics or magnitude of the cytokine and chemokine response during IAV infection, pulmonary concentrations of 24 cytokines and chemokines were measured at selected time-points. Pulmonary concentrations of pro-inflammatory cytokines and chemokines (e.g. IL-6, IFNγ, and CCL2) broadly increased in response to infection at 3 and 9 dpi, and then declined following control of viral replication at 14 dpi (**Fig 2A–2E** and **S1 Table**). The only chemokine that was significantly altered by testosterone treatment was CXCL1, which was found in greater concentrations in the lungs of testosterone-treated than testosterone-depleted male mice at 3 and 14, but not 9, dpi (**Fig 2E**; *p* < 0.05). Despite the known anti-inflammatory effects of testosterone [28–30], testosterone treatment did not alter pulmonary concentrations of either IL-10 or TGFβ during IAV infection (**Fig 2F** and **S1 Table**). Taken together, these data suggest that the improved outcome of IAV associated with testosterone is independent of substantial changes in the ‘cytokine storm’ during IAV infection, at least at the time-points selected.

### Testosterone alters the influx and contraction of pulmonary immune cells during the resolution of IAV infection

**Fig 3:**
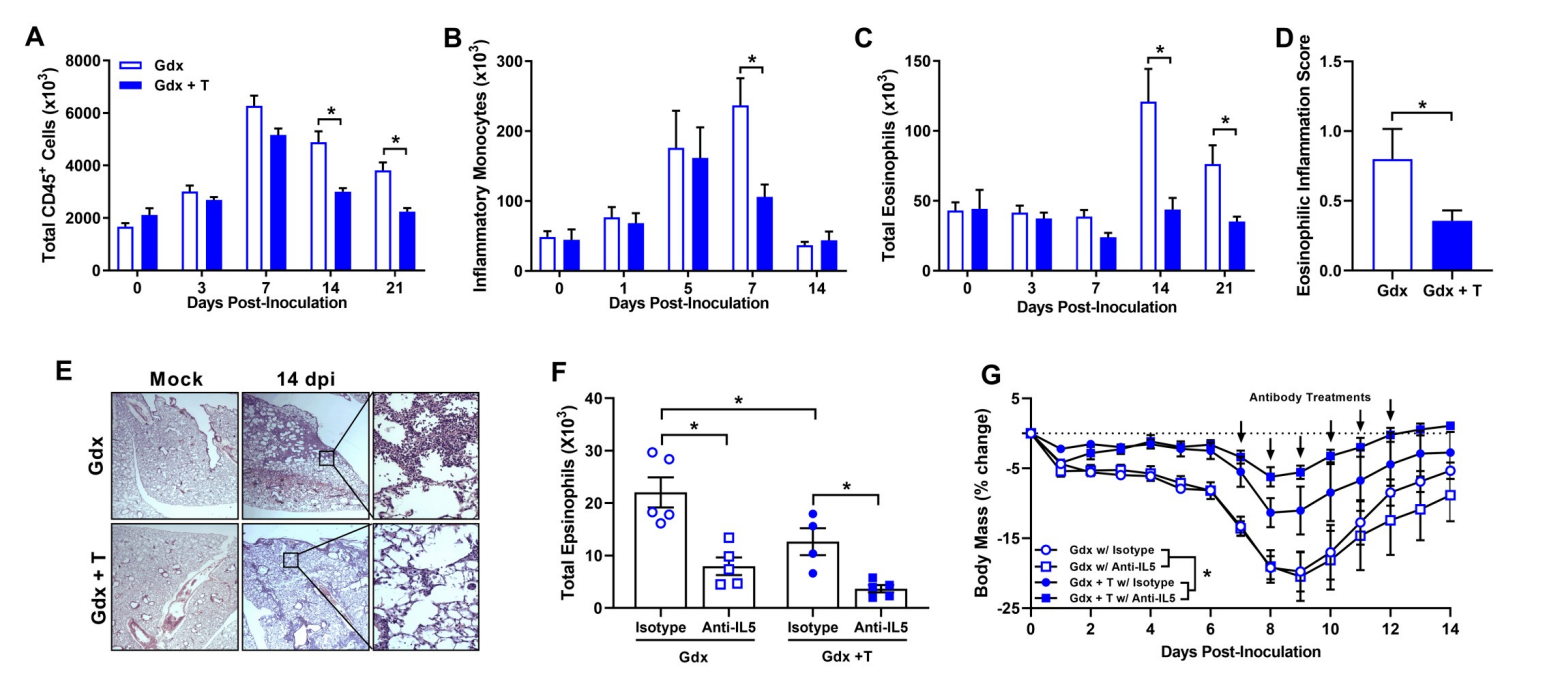
Testosterone treatment inhibits the influx of innate immune cells into the lungs during influenza A virus (IAV) infection. Adult male mice were gonadectomized and implanted with either testosterone (gdx + T) or placebo (gdx) containing capsules, and then inoculated with a sub-lethal dose of ma2009 H1N1 IAV or mock infected. At select days post inoculation (dpi), mice were euthanized, and pulmonary immune cells were quantified by flow cytometry (n = 6-10/treatment/time-point). Surface marker staining was used to identify numbers of CD45^+^ cells (A), inflammatory monocytes (B), and eosinophils (C). H&E stained lung sections collected at 14 dpi from ma2009 H1N1 IAV or mock infected mice (n = 5-7/treatment) were scored for eosinophilic inflammation (D), and representative photomicrographs (E) are shown. After IAV infection, eosinophils were depleted by IP injections of anti-IL-5 antibodies (anti-IL-5) or an isotype control antibody (isotype) daily from 7 to 12 dpi (n = 4-5/treatment). At 14 dpi, mice were euthanized, and lung tissue was collected to quantify the numbers of eosinophils (F), and mice were monitored daily for changes in body mass (G). Data represent means +/- SEM from two independent replications and significant differences between treatment groups are denoted by asterisks (*p < 0.05).

Differences in the numbers and kinetics of immune cells that influx into the lungs during infection can greatly impact IAV pathogenesis [31–33]. To test the hypothesis that testosterone affected immune cell recruitment into the lungs during IAV infection, total numbers of innate and adaptive immune cells were enumerated in the lungs of testosterone or placebo-treated gonadectomized male mice. The total number of leukocytes (i.e., CD45^+^ cells) in the lungs peaked at 7 dpi (i.e., during peak virus replication) and was followed by a greater decline in cell numbers in the lungs of testosterone-treated relative to placebo-treated males at 14 and 21 dpi (i.e., after control of virus replication) (**Fig 3A**; *p* < 0.05). Thus, the largest effect of testosterone on immune cell infiltrates was observed after control of virus replication.

To identify which immune cell types persisted in the lungs of testosterone-depleted males, we characterized the composition of pulmonary cellular infiltrates. During IAV infection, the numbers of interstitial macrophages, neutrophils, inflammatory monocytes, plasmacytoid dendritic cells, and conventional dendritic cells increased in both testosterone- and placebo-treated gonadectomized males, with the greatest numbers of innate immune cells in the lungs occurring at 7 dpi (**Table 1**). In contrast, the number of alveolar macrophages declined over the course of infection in all male mice (**Table 1**).

**Table 1:** Total numbers of pulmonary myeloid cells following IAV infection in gonadectomized mice treated with placebo (Gdx) or testosterone (Gdx + T).

Total numbers of cells (x10^3^)		0dpi	3dpi	7dpi	14dpi
**Alveolar macrophages**	**Gdx**	84.4±16.2	63.2±8.6	72.1±14.7	79.4±16.2
	**Gdx + T**	97.4±17.5	91.2±7.4	64.2±15.1	72.8±10.3
**Interstitial macrophages**	**Gdx**	46.3±6.0	188.2±32.1	896.0±97.6	295.1±38.0
	**Gdx + T**	56.7±11.9	106.6±9.8	812.7±54.2	170.3±18.1
**Neutrophils**	**Gdx**	**75.5±13.7**	**260.4±33.4**	487.0±45.3	208.4±45.8
	**Gdx + T**	243.8±40.7*	407.7±60.2*	487.9±36.6	203.4±37.7
**Plasmacytoid dendritic cells**	**Gdx**	41.4±8.1	170.3±28.9	792.5±79.3	282.9±34.7
	**Gdx + T**	53.7±12.8	101.6±12.8	672.5±66.3	145.7±14.4
**Dendritic cells**	**Gdx**	86.6±15.2	77.3±8.6	241.0±23.5	142.2±9.2
	**Gdx + T**	90.6±26.1	89.4±5.5	211.8±13.3	105.9±11.8

Data are presented as the mean +/- SEM from 2 independent experiments (n = 8-10/treatment/timepoint) and significant differences between treatment groups are bolded and denoted by asterisks (*P < 0.05).

The only innate immune cell populations affected by testosterone treatment in males were neutrophils, inflammatory monocytes, and eosinophils. The number of pulmonary neutrophils was transiently greater at baseline (0 dpi) and 3pi, but not at other dpi, in gonadectomized males that were treated with testosterone as compared with those treated with placebo (**Table 1**; *p* < 0.05). Following IAV infection, the contraction of inflammatory monocyte numbers in the lungs was accelerated in testosterone-treated males compared with placebo-treated males during peak disease (i.e., at 7 dpi; **Fig 3B**; *p* < 0.05). In contrast, a significant influx of eosinophils into lungs occurred in gonadectomized males treated with placebo, but not testosterone, after clearance of detectable virus from the lungs (i.e., 14 and 21 dpi) (**Fig 3C**; *p* < 0.05).

The influx of eosinophils into the lungs of testosterone-depleted males during the resolution phase of infection may be indicative of eosinophilic pneumonia [34], which could contribute to the greater pulmonary inflammation analyzed previously by histology in testosterone-depleted male mice [22]. To test this hypothesis, histopathological scoring for specific markers of eosinophilic inflammation was performed on H&E stained lung sections. Fourteen dpi, histologic evidence of eosinophilic pulmonary inflammation was greater in placebo-treated compared with testosterone-treated males, which was characterized by the accumulation of eosinophils within the interstitium and alveolar spaces (**Fig 3D and Fig 3E**; *p* < 0.05).

To determine whether the inhibition of pulmonary eosinophil accumulation was mediating testosterone-dependent protection against severe IAV, mice were gonadectomized, treated with either testosterone or placebo, infected with IAV, and treated daily with either IL-5 neutralizing antibodies or isotype control antibodies between 7 and 12 dpi. Fourteen dpi, the numbers of pulmonary eosinophils were significantly reduced in testosterone-treated compared with placebo-treated males, with IL-5 neutralizing antibody treatment significantly reducing the numbers of pulmonary eosinophils in all males, regardless of testosterone status (**Fig 3F**; *p* < 0.05). Reduction of pulmonary eosinophils with anti-IL-5 antibody treatment did not protect testosterone-depleted males against IAV (**Fig 3G**; *p* < 0.05). These data suggest that the inhibition of pulmonary eosinophil accumulation does not directly mediate the protective effects of testosterone on IAV pathogenesis and is likely caused by other testosterone-mediated changes in pulmonary inflammation.

In addition to affecting innate immune cells, testosterone is associated with shifts in the numbers, activities, and differentiation of CD4^+^ T cells in experimental models of allergy and autoimmune disease [28, 35–37]. To test the hypothesis that testosterone improved the outcome of IAV infection by shifting populations of CD4^+^ T cells, helper T cell type 1 (Th1), type 2 (Th2), type 17 (Th17), and regulatory T (Treg) cells were quantified at several time points before and during IAV infection. Peak numbers of total CD4^+^ T cells, Th1, Th2, Th17, and Treg cells occurred in both testosterone-depleted and -replete males at 9 dpi, followed by a reduction of cell numbers at 14 dpi (**Table 2**). The contraction of total CD4^+^ T cell numbers in the lungs was significantly slower in testosterone-depleted as compared with replete males at 14 and 21 dpi (**Table 2**; *p* < 0.05). There was no effect of testosterone treatment on the numbers of IAV-specific Th1, Th2, or Th17 cells at any time point examined (**Table 2**). In contrast with previous reports of testosterone-induced expansion of Treg cell numbers [37], Treg cell numbers were greater in the lungs of placebo-treated relative to testosterone-treated gonadectomized males, but only at 21 dpi (**Table 2**; *p* < 0.05). These data suggest that virus-specific CD4^+^ T cells are not the primary cell type mediating the protective effects of testosterone during IAV infection of male mice.

**Table 2:** Total numbers of pulmonary CD4^+^ T cells following IAV infection in gonadectomized mice treated with placebo (Gdx) or testosterone (Gdx + T).

Total numbers of cells (x10^3^)		0dpi	5dpi	9dpi	14dpi	21dpi
**Total CD4**^**+**^ **T cells**	**Gdx**	367.1±37.6	538.7±61.7	1740.5±248.2	838.5±45.9*	611.7±61.7*
	**Gdx + T**	289.8±47.3	498.8±69.3	1487.7±135.3	**434.6±39.0**	**281.1±40.8**
**Th1 cells**	**Gdx**	3.3±1.2	1.3±0.5	14.8±3.0	7.1±2.7	10.4±1.5
	**Gdx + T**	1.7±0.5	3.0±1.8	19.1±5.8	7.6±1.3	7.1±1.4
**Th2 cells**	**Gdx**	6.4±2.0	5.3±1.3	33.4±5.3	24.5±1.3	17.3±3.4
	**Gdx + T**	8.3±1.7	6.0±1.7	36.3±7.4	19.3±2.0	8.5±0.9
**Th17 cells**	**Gdx**	4.5±1.5	10.2±4.1	24.4±2.8	7.5±1.3	6.0±0.9
	**Gdx + T**	6.0±1.2	8.3±2.4	23.0±3.1	7.7±1.3	3.7±1.0
**Regulatory T Cells**	**Gdx**	5.3±1.2	17.5±2.1	28.2±9.6	13.8±2.3	16.3±1.4*
	**Gdx + T**	3.4±0.8	12.5±1.4	30.5±7.9	12.7±3.6	**7.3±0.8**

Helper T cell type 1 (Th1), Th2, and Th17 cells were classified as expressing IFNγ, IL-4, or IL-17A respectively in response to virus-specific peptide stimulation. Regulatory T cells were classified as CD4^+^ CD25^+^ and FoxP3^+^ cells. Data represent means +/- SEM from 2 independent experiments (n = 6-12/treatment/timepoint) and significant differences between treatment groups are bolded and denoted by asterisks (*P < 0.05).

**Fig 4:**
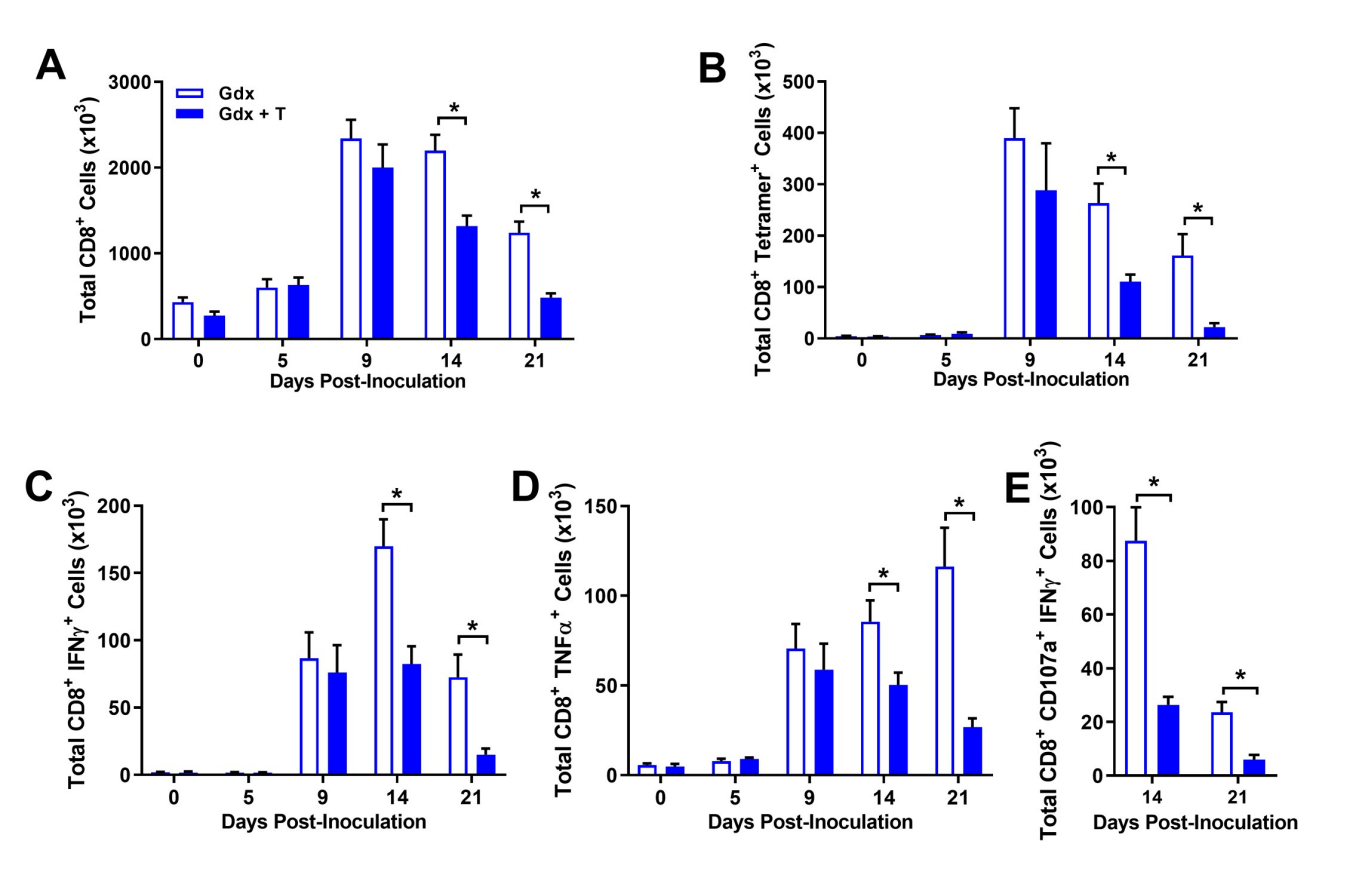
Testosterone treatment reduces numbers and activity of virus-specific CD8^+^ T cells following control of viral replication. Adult male mice were gonadectomized and implanted with either testosterone (gdx + T) or placebo (gdx) containing capsules, and then inoculated with a sub-lethal dose of ma2009 H1N1 IAV or mock infected. At select days post inoculation (dpi), mice were euthanized, and pulmonary immune cells were quantified by flow cytometry (n = 7-14/treatment/time-point). Surface marker and intracellular staining was used to identify numbers of total CD8^+^ T cells (A), ma2009 H1N1-specific CD8^+^ T cells (B), CD8^+^ T cells producing IFNγ (C) or TNFα (D) in response to *ex vivo* H1N1-specific peptide stimulation, and poly-functional CD8^+^ T cells expressing both CD107a and IFNγ (E) following *ex vivo* H1N1-specific peptide stimulation. Data represent means +/- SEM from two independent replications and significant differences between treatment groups are denoted by asterisks (*p < 0.05).

Virus-specific CD8^+^ T cells are beneficial for the killing of IAV-infected cells but can also be detrimental to the host by causing immunopathology [38, 39]. Total CD8^+^ as well as IAV-specific CD8^+^ T cells influxed into the lungs at 9 dpi (**Fig 4A and 4B**), which corresponded with peak virus titers (**Fig 1F**). Testosterone had no effect on either the induction or peak magnitude of total or virus-specific CD8^+^ T cells measured in the lungs; the contraction of these cells, however, following the clearance of detectable virus (14 and 21 dpi) was significantly improved in testosterone-treated as compared with placebo-treated gonadectomized males (**Fig 4A and 4B**; *p* < 0.05 in each case). To determine if testosterone suppressed the activity of virus-specific CD8^+^ T cells, we assessed cytokine production following *ex vivo* stimulation with ma2009 H1N1-specific peptide and observed that the number of CD8^+^ T cells producing either IFNγ or TNFα was significantly reduced in testosterone-treated males relative to testosterone-depleted males after virus had been cleared (14 and 21 dpi) but not during peak virus titers (9 dpi; **Fig 4C** and **Fig 4D**; *p* < 0.05). To further assess whether testosterone affected the functionality of IAV-specific CD8^+^ T cell we used CD107a as a marker for degranulation in response to *ex vivo* ma2009 H1N1-specific peptide stimulation. After virus had been cleared from the lungs, the contraction in the numbers of CD8^+^ T cells staining positive for both surface expression of CD107a and production of IFNγ was delayed in the lungs of testosterone-depleted relative to testosterone-replete male mice (**Fig 4E**; *p* < 0.05). The effects of testosterone on the contraction of IAV-specific CD8^+^ T cells after virus had been cleared from the lungs, was specific to the site of virus replication and not observed in either the spleen or the mediastinal lymph nodes (i.e. pulmonary draining lymph nodes; **Table 3**). Taken together, these data suggest that testosterone may improve the outcome of primary IAV infection by dampening immunopathology caused by pulmonary virus-specific CD8^+^ T cell populations.

**Table 3:** Total numbers of CD8^+^ T cells in the mediastinal lymph nodes and spleens of gonadectomized mice treated with placebo (Gdx) or testosterone (Gdx + T) following IAV infection.

		Mediastinal Lymph Nodes		Spleen	
Total # of cells (x10^3^)		14dpi	21dpi	14dpi	21dpi
**Total CD8**^**+**^ **T cells**	**Gdx**	1687.8±128.6	1425.7±150.8	8030.1±957.2	6413.7±405.8
	**Gdx + T**	1442.9±166.5	1388.1±263.8	7949.4±599.9	7943.4±614.6
**Tetramer**^**+**^ **CD8**^**+**^ **T cells**	**Gdx**	22.0±6.5	12.1±1.4	75.1±10.2	77.6±17.3
	**Gdx + T**	13.3±2.4	9.5±1.2	94.3±17.9	77.2±10.9
**IFNγ**^**+**^ **CD8**^**+**^ **T cells**	**Gdx**	7.9±1.2	6.8±2.0	69.2±18.0	35.2±8.0
	**Gdx + T**	6.7±1.5	3.7±1.0	70.8±13.0	36.2±12.5
**TNFα**^**+**^ **CD8**^**+**^ **T cells**	**Gdx**	55.6±3.1	20.6±6.7	256.7±16.8	93.7±41.5
	**Gdx + T**	47.8±5.2	18.0±4.8	308.7±43.0	103.7±39.4

Data are presented as the mean +/- SEM from 2 independent experiments (n = 5/treatment/timepoint).

### Testosterone creates a local environment to promote the contraction of CD8^+^ T cells following control of IAV replication

**Fig 5:**
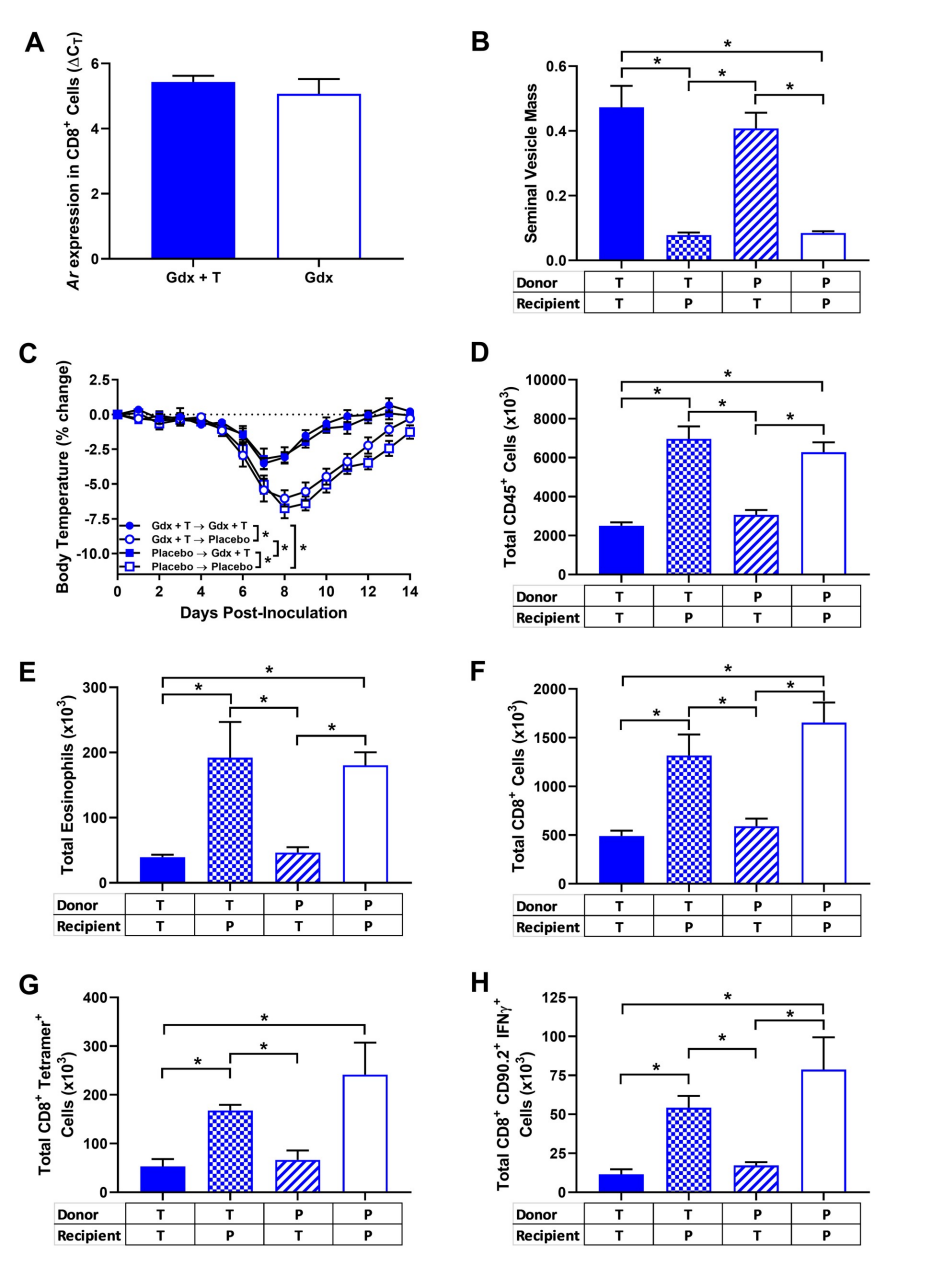
Secondary effects of testosterone on the contraction of CD8^+^ T cell populations following control of viral replication. Adult male TCR-Ova mice were gonadectomized and implanted with capsules containing testosterone or placebo prior to infection with a sub-lethal dose of WSN-Ova_1_ H1N1 IAV (n = 5/treatment group). At 14 days post inoculation (dpi), mice were euthanized, CD8^+^ T cells were isolated by negative selection, and splenic mRNA was measured and normalized to GAPDH using the ΔCT method (A). Adoptive transfer experiments were performed, and adult male TCR-Ova and CD90.1 mice were gonadectomized and implanted with capsules containing either placebo (gdx) or testosterone (gdx + T). Splenic CD8^+^ T cells were isolated from placebo- or testosterone-treated donor TCR-Ova mice by negative selection purification, and adoptively transferred by tail vein injection into either placebo- or testosterone-treated male CD90.1 recipient mice. Mice were then infected by intranasal inoculation with a sub-lethal dose of WSN-Ova_1_ H1N1 IAV, and seminal vesicle mass was quantified as the percentage of total body mass (B; n = 6-7/treatment). Mice were monitored daily for changes in body temperature (C). At 14 dpi, mice were euthanized, and lung tissue was collected to quantify the numbers of CD45^+^ cells (D), eosinophils (E), total CD8^+^ T cells (F), Ova-specific CD8^+^ T cells (G), and adoptively transferred CD90.2^+^ CD8^+^ T cells producing IFNγ in responses to OVA-specific peptide stimulation (H) were quantified by flow cytometry (n = 6-7/treatment). Data represent means +/- SEM from two independent replications and significant differences between treatment groups are denoted by asterisks (*p < 0.05).

Testosterone can act both directly and indirectly to alter the biological activities of T cells [28, 40, 41]. For testosterone to have direct effects on the contraction of CD8^+^ T cells during IAV infection, AR expression would need to occur within these cells. Consistent with previous reports [28, 42–44], *Ar* mRNA was expressed in enriched splenic CD8^+^ T cells from both testosterone-depleted and testosterone-replete male mice, with no effect of testosterone treatment on *Ar* mRNA expression (**Fig 5A**).

Because *Ar* mRNA was expressed by splenic CD8^+^ T cells, we hypothesized that testosterone could condition these cells to induce contraction following control of viral replication. To test this hypothesis, TCR-Ova donor mice and CD90.1 recipient mice were gonadectomized and implanted with capsules containing either testosterone or placebo. Enriched splenic CD8^+^ T cells from naïve TCR-Ova mice were then adoptively transferred into either placebo- or testosterone-treated CD90.1 recipient mice prior to infection with WSN-Ova_1_ H1N1 IAV. Seminal vesicle mass was greater in testosterone-treated recipient mice relative to placebo-treated recipient mice for all treatment groups (**Fig 5B**; *p* < 0.05). Recipient males that were gonadectomized and treated with testosterone prior to IAV infection were protected against IAV and experienced similar levels of morbidity (i.e., temperature loss), regardless of the hormonal milieu of the donor transferred CD8^+^ T cells (**Fig 5C**; *p* < 0.05). Testosterone treatment of recipient mice accelerated the contraction of total numbers of CD45^+^ leukocytes, eosinophils, CD8^+^ T cells, and WSN-Ova_1_ H1N1-specific CD8^+^ T cell relative to testosterone-depleted recipient male mice at 14dpi (**Fig 5D–5G**; *p* < 0.05 in each case), regardless of the hormonal environment of the donor transferred CD8^+^ T cells. Moreover, the contraction of adoptively transferred CD90.2^+^ CD8^+^ T cells producing IFNγ in response to *ex vivo* Ova-specific peptide stimulation was accelerated in testosterone-treated recipient mice at 14 dpi (**Fig 5H**; *p* < 0.05) relative to testosterone-depleted recipient mice, irrespective of the donor mouse treatment. These data demonstrate that accelerated contraction of virus-specific CD8^+^ T cells occurs in an environment that contains testosterone. These data further suggest that the effects of testosterone on contraction of virus-specific CD8^+^ T is a secondary, rather than primary, effect of testosterone treatment.

### The protective effects of testosterone during IAV infection are dependent on androgen receptor signaling

**Fig 6:**
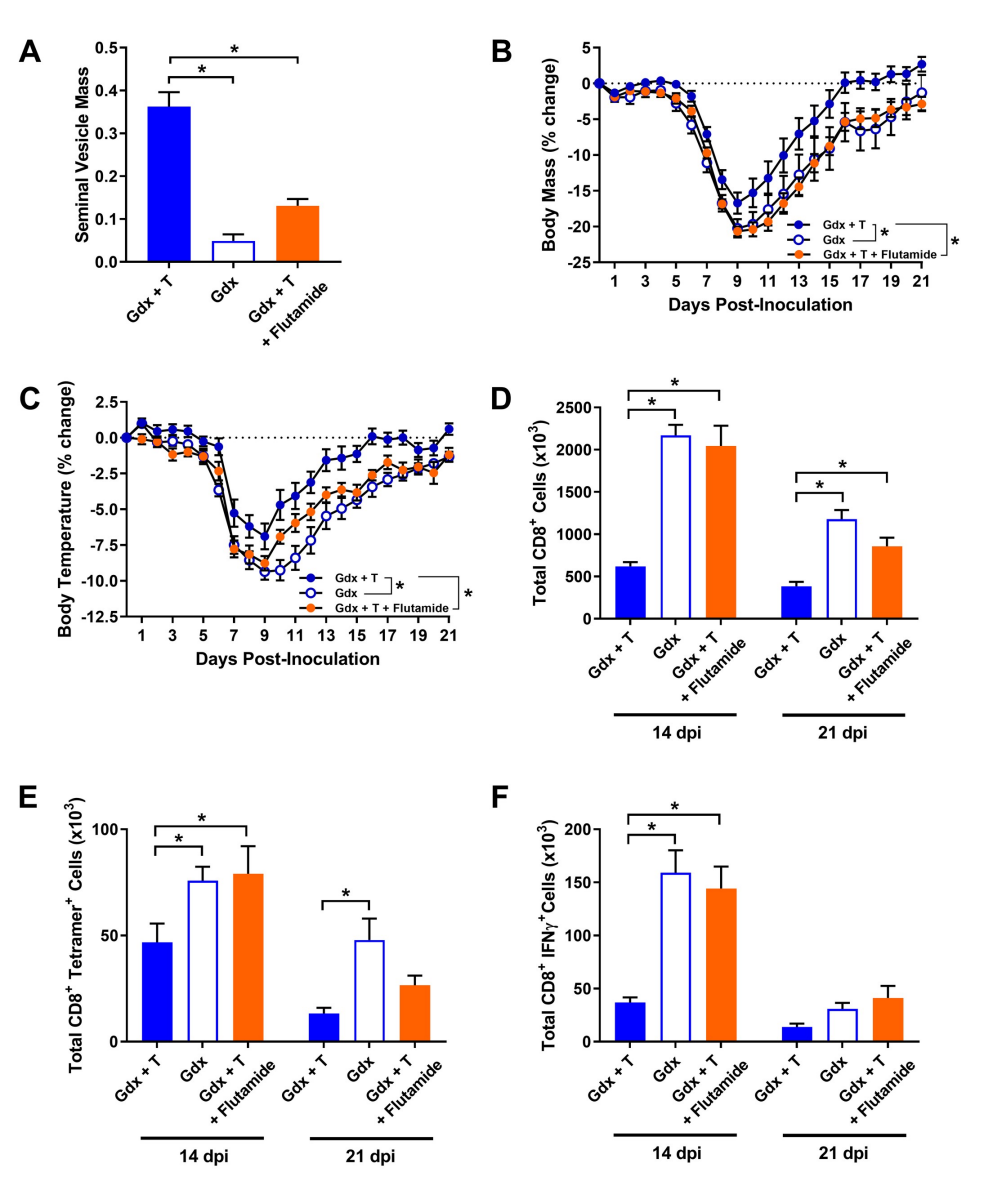
The androgen receptor antagonist, flutamide, inhibits the protective effects of testosterone treatment on influenza A virus (IAV) pathogenesis. Adult male mice were gonadectomized and implanted with capsules containing placebo (gdx), testosterone (gdx + T), or flutamide + testosterone (flutamide + T), and seminal vesicle mass was quantified as the percentage of total body mass (A; n = 9/treatment). Following intranasal inoculation with a sub-lethal dose of ma2009 H1N1 IAV, mice were monitored daily for changes in body mass (B) and body temperature (C) for 21 days post inoculation (dpi; n = 12-15/treatment). At 14- and 21-dpi, the total numbers of CD8^+^ T cells (D), H1N1-specific CD8^+^ T cell numbers (E), and the number of CD8^+^ T cells producing IFNγ in response to *ex vivo* H1N1-specific peptide stimulation (F) were quantified by flow cytometry (n = 8-10/treatment/time-point). Data represent means +/- SEM from two independent replications and significant differences between treatment groups are denoted by asterisks (*P < 0.05).

Testosterone can be metabolized in tissues, converted into estradiol, and signal through estrogen receptors (ERs) [45]. Estradiol signaling through ERα can dampen inflammation and improve the outcome of IAV infection, at least in female mice [21, 46, 47]. To determine whether the protective effects of testosterone during IAV infection in male mice were caused by signaling through AR or ER, male mice were gonadectomized and implanted with capsules containing either testosterone, placebo, or a combination of testosterone and the AR antagonist, flutamide [48]. Seminal vesicle mass was used as a biomarker to confirm AR inhibition by flutamide and was significantly reduced in males that received testosterone + flutamide treatment as compared with males that received testosterone alone (**Fig 6A**; *p* < 0.05). During IAV infection, flutamide treatment inhibited the protective effects of testosterone on morbidity (**Fig 6B and 6C**; *p* < 0.05). To assess whether the testosterone-induced changes in CD8^+^ T cell numbers and activity were also AR-dependent, we evaluated the effects of flutamide on the contraction of CD8^+^ T cells during the resolution phase of infection. Co-treatment of flutamide and testosterone, similar to placebo treatment, resulted in significantly greater numbers of total CD8^+^ T cells, ma2009 H1N1-specific CD8^+^ T cell, and virus-specific CD8^+^ T cells producing IFNγ in response to H1N1 specific peptide stimulation at 14 and 21 dpi as compared with testosterone treatment alone (**Fig 6D–6F**; *p* < 0.05).

**Fig 7:**
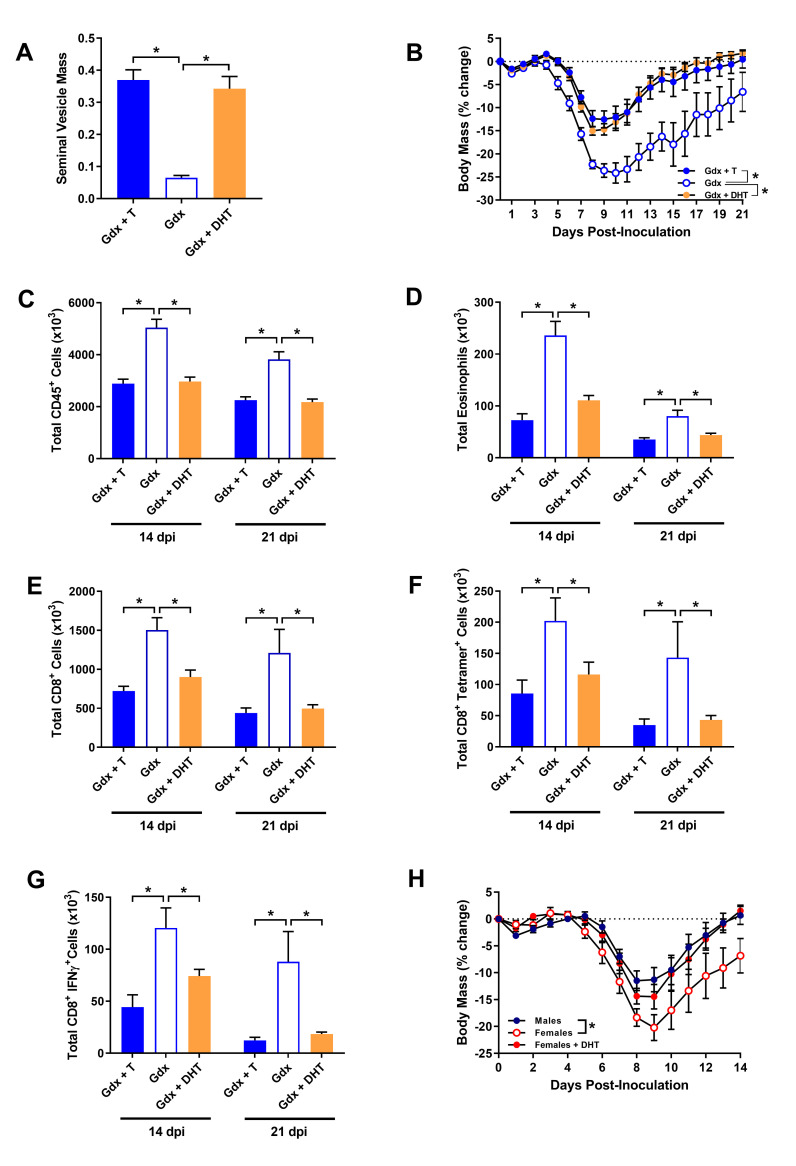
The non-aromatizable androgen, dihydrotestosterone (DHT) mimics the protective effects of testosterone on influenza A virus (IAV) pathogenesis in male and female mice. Adult male mice were gonadectomized and implanted with capsules containing testosterone (gdx + T), DHT (gdx + DHT), or placebo (gdx), and seminal vesicle mass was quantified as the percentage of total body mass (A; n = 6-8/treatment). Following intranasal inoculation with a sub-lethal dose of ma2009 H1N1 IAV, mice were monitored daily for changes in body mass (B) for 21 days post inoculation (dpi; n = 13-15/treatment). At 14 and 21 dpi, the total numbers of CD45^+^ cells (D), eosinophils (E), CD8^+^ T cells (F), H1N1-specific CD8^+^ T cell numbers (G), and the number of CD8^+^ T cells producing IFNγ in response to *ex vivo* H1N1-specific peptide stimulation (H) were quantified by flow cytometry (n = 8-12/treatment/time-point). Female mice were left gonadally intact prior to implantation with capsules containing placebo (female) or DHT (female + DHT), infected with IAV, and monitored daily for changes in body mass (H) for 14 dpi for comparison with gonadally intact male mice (n = 9-10/treatment/sex). Data represent means +/- SEM from two independent replications and significant differences between treatment groups are denoted by asterisks (*p < 0.05).

Because flutamide can alter T cell function through off-target GABA-A receptor signaling [49], we sought to confirm the effects of AR signaling on IAV pathogenesis by using the non-aromatizable androgen, dihydrotestosterone (DHT) (i.e., an androgen that cannot be converted into estradiol). Treatment of gonadectomized males with DHT significantly increased seminal vesicle mass relative to placebo-treated males, to a mass consistent with testosterone-treated males (**Fig 7A**; *p* < 0.05). Males that were gonadectomized and treated with DHT prior to IAV infection were protected against IAV and experienced a similar level of morbidity (i.e., body mass loss) as testosterone-treated males, which was collectively better than placebo-treated mice (**Fig 7B**; *p* < 0.05). Consistent with testosterone, DHT inhibited the influx of eosinophils into lungs, and accelerated the contraction of total numbers of leukocytes (i.e., CD45^+^ cells), total numbers of CD8^+^ T cells, ma2009 H1N1-specific CD8^+^ T cell numbers, and the number of CD8^+^ T cells producing IFNγ in response to *ex vivo* H1N1-specific peptide stimulation relative to testosterone depleted male mice at 14 and 21 dpi (**Fig 7C–7G**; *p* < 0.05 in each case). Taken together, these data demonstrate that the protective effects of testosterone on IAV pathogenesis are dependent on AR signaling, which creates a pulmonary environment conducive to reduced pulmonary inflammation.

Female mice have lower testosterone concentrations, and like hypogonadal males, experience more severe disease following IAV infection relative to age-matched young adult males [21, 23]. To determine if the protective effects of AR signaling on IAV pathogenesis were sex-dependent, female mice were implanted with capsules containing either DHT or placebo prior to IAV infection. Consistent with previous data [21, 23], gonadally intact female mice treated with placebo experienced increased IAV morbidity (i.e., body mass loss) relative to gonadally intact males (**Fig 7H**; *p* < 0.05). Female mice treated with DHT were, however, protected against IAV and experienced similar levels of morbidity (i.e. body mass loss) to gonadally intact males (**Fig 7H**). These data demonstrate that the protective effects of AR signaling on the severity of IAV infection are not limited to males.

## Discussion

Inflammatory immune responses, including cytokine production and the cell-mediated activities of both myeloid and lymphoid cells, are required to control IAV infection, but if improperly regulated can contribute to tissue damage and severe outcomes [19, 33, 50–52]. In the current and previous studies [21, 22], androgens, including testosterone and DHT, in male mice reduce the severity of IAV infection by promoting the resolution of pulmonary inflammation rather than by affecting viral replication. The improved resolution of IAV-induced inflammation [22] in androgen-treated males was not caused by suppression of the cytokine storm, but rather by accelerated contraction of pulmonary Ly6C^+^ monocytes during peak inflammation and the mitigation of pulmonary CD8^+^ T cells and eosinophils after virus was cleared. The effect of androgens on pulmonary leukocyte activity was dependent on AR signaling in the lungs, which created a pulmonary environment that reduced the numbers and activities of these cells in the lungs following IAV infection.

We and others [21, 23] have shown that males experience less severe disease and recover faster from IAV than females. Data from the current study and others [22, 23] illustrate that androgens limit pulmonary inflammation during IAV infection, thus maintaining greater tolerance during infection in males and even females. In addition to reduced inflammation, males also repair damaged tissue faster than females which is mediated by greater production of epidermal growth factor amphiregulin in males than females [23]. Testosterone does not regulate production of amphiregulin; therefore, elevated levels of both testosterone and amphiregulin contribute to improved IAV outcomes in males than females.

In the current study, the depletion of testosterone resulted in the accumulation of inflammatory monocytes during peak virus replication and eosinophils in the lungs following control of viral replication, which was unexpected given the lack of observed changes in pulmonary concentrations of IL-5, IL-13, and exotoxin. Eosinophils are androgen responsive despite the absence of AR expression [53–55], with testosterone-mediated differences in eosinophilic airway responses instead being attributed to the actions of Type II innate lymphoid cells (ILC2s) [56, 57]. Though not evaluated in this study, androgens have been shown to inhibit the maturation of ILC2s, while decreasing IL-5 production and eosinophilic responses in murine models of airway inflammation [56, 57]. Although the precise role of eosinophils in the immune response to IAV is unclear, previous studies in mice show accumulation of eosinophils in the lungs following control of viral replication [58, 59]. In the current study, depletion of eosinophils during the later stages of infection did not reduce morbidity in testosterone-depleted males, suggesting that the protective effects of testosterone on IAV pathogenesis are not directly mediated by effects on eosinophils. Whether the accumulation of eosinophils during the resolution phase of infection represents the activation of type 2 tissue repair responses [60], or a pathological response contributing to immunopathology warrants further study.

Monocytes play a critical role in the control of respiratory virus infection through the coordination of the immune response (i.e., cytokine and chemokine secretion) in addition to functioning as tissue progenitor cells for several monocyte-derived DC and macrophage populations [53]. Despite this beneficial role, exaggerated or improperly regulated inflammatory monocyte responses to IAV infection contribute to pulmonary damage and adverse clinical outcomes [61, 62]. Consistent with these observations, in the present study depletion of testosterone delayed the contraction of pulmonary Ly6C^+^ inflammatory monocytes early during IAV infection. Conversely, depletion of testosterone did not alter pulmonary concentrations of Ly6C^+^ monocyte-associated cytokines or chemokines (e.g., CCL2 and TNFα) [63]. Future studies will need to define the mechanism by which testosterone depletion promotes the persistence of these cells and determine if they are the primary mediator of testosterone-dependent differences in IAV outcomes.

In response to other inflammatory diseases, including experimental autoimmune encephalomyelitis, testosterone is associated with an expansion of Th2 and Th17 cell populations and suppression of Th1 activity [37, 64, 65]. During IAV pathogenesis, testosterone treatment of males accelerated the contraction of total pulmonary CD4^+^ numbers but did not lead to shifts in the differentiation of virus-specific CD4^+^ T helper cell populations. Diversification of the peptide pool used to assess IAV-specific CD4^+^ T cells may yield differential effects of testosterone on CD4^+^ T cell subsets as in the current study a single peptide was used for stimulation. Furthermore, although testosterone treatment can promote the expansion in numbers and activation of Treg cells in murine models of inflammation [37, 66, 67], there was only a transient effect of testosterone on Treg cells during IAV infection. Whether this represents differences in the polarizing effects of viral infection versus other inflammatory states should be considered.

During IAV infection, CD8^+^ T cells also play a critical role in the control of IAV infection through the production of cytokines and the killing of virus-infected cells [68]. Improper regulation or prolonged activation of virus-specific CD8^+^ T cell responses, however, can also cause immunopathology and severe pulmonary tissue damage [38, 39]. Both in humans and mice, testosterone alters the numbers, cytokine production, and proliferative potential of CD8^+^ T cells [16, 41]. Consistent with these observations, androgens, including testosterone, accelerated the contraction of virus-specific CD8^+^ T cells in the lungs, but not in the spleen or mediastinal lymph nodes. The significance of this tissue specific effect is unknown, but whether these effects of testosterone on virus-specific CD8^+^ T cells involve activation-induced cell death, inhibitory pathways, interactions with tissue-specific cell types, including TNF/iNOS-producing DCs [69], or changes in the establishment of tissue resident memory cell populations warrants future study.

The expression of *Ar* in enriched splenic CD8^+^ T cell populations suggested that testosterone might be acting directly on these CD8^+^ T cell to mitigate IAV pathogenesis. Adoptive transfer studies were conducted and revealed that the presence of testosterone in the recipient mice was a better predictor of IAV outcome and contraction of virus-specific CD8^+^ T cells than the presence of testosterone in the donor mice. These data suggest that testosterone does not condition virus-specific CD8^+^ T cells to induce intrinsic changes in these cells via AR signaling. Instead, testosterone induces changes in these cells that are conditional on the presence of testosterone in the local environment in which they reside. Whether testosterone treatment results in functional AR signaling in CD8^+^ T cells was not assessed in the current study. As previous reports are inconsistent with regards to the degree and nature of AR signaling in mature CD8^+^ T cells [40, 44, 70], in vitro co-culture experiments may prove useful in elucidating whether testosterone acts directly on these cells to improve the outcome of IAV infection.

Given the widespread expression of AR both in immune cells and epithelial cells in the lung [53, 71], testosterone may be acting indirectly on virus-specific CD8^+^ T cells, through interactions with other cells to promote their contraction. One such cell type may be the Ly6C^+^ inflammatory monocytes which persisted in the lungs of testosterone depleted males, and have recently been shown to promote the persistence of lung resident memory CD8^+^ T cells following respiratory virus infection [72]. Similarly, suppression of Ly6C^+^ inflammatory monocyte derived TNF/iNOS-producing DCs reduces tissue specific proliferation and survival of antigen-specific CD8^+^ effector T cells [69]. Whether testosterone acts through inflammatory monocytes to alter the generation and persistence of virus-specific CD8^+^ T cell populations warrants future study.

Testosterone can be metabolized by aromatase into estradiol to signal through ERα, which can dampen inflammation and improve the outcome of IAV infection in females [21, 45–47]. Moreover, in male mice gonadectomized prior to the onset of puberty, castration-mediated protection against lethal IAV infection is reversed by testosterone treatment and subsequent conversion to estradiol, but not by treatment with non-aromatizable DHT [73]. In the present study, the protective effects of testosterone on IAV pathogenesis were dependent on AR signaling in the lungs. The discordant findings regarding the impact of androgens on IAV pathogenesis in prepubertal versus adult mice likely represent differences in the developmental effects of sex steroid signaling.

We have previously demonstrated that low concentrations of testosterone, whether age-associated, surgically-induced, or driven by biological sex, causes delayed resolution of pulmonary inflammation following, but not prior to, the control of viral replication [22, 23]. In the present study, delayed resolution of disease was associated with the persistence of total leukocytes and pulmonary CD8^+^ T cells following control of viral replication and an influx of eosinophils into the lungs at 14 dpi. Despite this temporal correlation, neither the depletion of eosinophils nor adoptive transfer of CD8^+^ T cells could reverse the ability of testosterone to protect males against IAV, suggesting that the effect of testosterone on these cell types is secondary to effects on other immune mediators. Testosterone promoted the early contraction of Ly6C^+^ inflammatory monocytes which correlated with the early divergence in morbidity (i.e., clinical severity and body mass) between testosterone-depleted and testosterone replete males during peak pulmonary inflammation. From these data, we hypothesize that androgen-induced changes in inflammatory monocytes maybe the cause of testosterone-mediated protection during IAV and the downstream reduction of eosinophilic inflammation and pulmonary CD8^+^ T cell persistence is a secondary consequence to changes in inflammatory monocyte numbers.

The impact of testosterone on infectious disease outcomes involves many cell types and responses. While our work has shown androgen-induced changes in immune function to be protective, these same immunological changes can be detrimental in other instances, including with amoebic infection, in which treatment with testosterone increases the severity of infection at least in part through inhibition of IFNγ production by natural killer T cells [74–76]. When disease following infection is caused by the inability to control the pathogen, then androgens, like testosterone, are detrimental. Conversely, when disease following infection is largely attributable to immunopathology [19, 53], the immunomodulatory effects of testosterone, and more specifically, AR signaling, are likely protective. These data suggest that testosterone confers protection during IAV infection by modulating the immune response and suggest that androgens may have therapeutic potential in female and hypogonadal male populations.

## Materials and methods

### Ethical statement

All experiments were performed in compliance with the standards outlined in the National Research Council’s Guide to the Care and Use of Laboratory Animals. All animal procedures were approved by the Johns Hopkins Animal Care and Use Committee (MO18H262). All efforts were made to minimize animal suffering.

### Animals

Adult (7–8 weeks old) male and female C57BL/6 mice were purchased from Charles River. For adoptive transfer experiments, male and female TCR-Ova (C57BL/6-Tg(TcraTcrb) 1100MjbJ/J) and CD90.1 (B6.PL-*Thy1* ^*a*^/CyJ) mice were purchased from The Jackson Laboratory as breeding pairs and bred in house to obtain male offspring. All mice were housed at 3–5 animals per microisolator cage under standard BSL-2 housing conditions and given food and water *ad libitum*.

### Gonadectomy and hormone manipulation

Adult (8 week old) male mice were anesthetized by intra-peritoneal (IP) inoculation with a ketamine (80 mg/kg) and xylazine (8 mg/kg) cocktail and the testes were removed bilaterally as described previously [21]. Following two weeks recovery, silastic tubing capsules (inner diameter-0.04", outer diameter- 0.085"; HelixMark) containing crystalline testosterone propionate (7.5 mm; Sigma), crystalline 4,5α-Dihydrotestosterone (5.0 mm; Sigma), or nothing were implanted subcutaneously [77]. For flutamide studies, capsules were prepared as above (2 x 15.0 mm; Sigma) but were implanted at the time of gonadectomy. Adult (8 week old) female mice were left gonadally intact prior to capsule implantation. All capsules were sealed with 2.5 mm of medical adhesive (Factor II, A-100) and incubated at 37°C overnight in sterile saline solution prior to implantation.

### Virus infection and quantification

Mouse-adapted A/California/4/09 (ma2009; H1N1; generated by Dr. Andrew Pekosz using a published sequence) [78] or recombinant A/WSN/33 virus containing OVA_257-264_ (SIINFEKL) peptide in the neuraminidase protein (H1N1; WSN-Ova_1_) [79], were used in all experiments. Mice were anesthetized and infected by intranasal inoculation with ma2009 or WSN-Ova_1_ H1N1 virus (ma2009 = 0.1 MLD_50_; WSN-OVA_1_ = 0.4 MLD_50_) diluted in 30μl of DMEM or mock infected with 30μl DMEM. For virus quantification, log_10_ dilutions of lung homogenate were plated onto Madin-Darby canine kidney (MDCK) cell monolayers in replicates of 6 for 5 days at 32°C. Cells were stained with naphthol blue black (Sigma Aldrich) and scored for cytopathic effect. The 50% tissue culture infectious dose (TCID_50_) was calculated using the Reed-Muench method and was used to back titer all viral inoculums.

### Sample collection and testosterone quantification

Following infection, rectal temperature and body mass were recorded daily out to 21 days post inoculation (dpi), and clinical disease scores were recorded at select time-points as described previously [22]. For terminal studies, mice were euthanized at select time-points and plasma, whole lungs, spleen, and mediastinal lymph nodes (MLN) were collected. Seminal vesicles were also collected, and mass was recorded as a bio-marker for androgen activity. Total testosterone concentration was quantified in plasma collected at 21 dpi by commercial ELISA kit according to the manufacturer’s instructions (IBL America). To prevent sample degradation, care was taken to limit light and thermal exposure of plasma samples prior to testosterone quantification.

### Pulmonary cytokine and chemokine quantification

Snap-frozen lung tissue was homogenized in DMEM supplemented with 1% L-glutamine (Gibco), and 1% penicillin-streptomycin (Gibco) and centrifuged to remove cellular debris. Supernatants were collected and Eotaxin, G-CSF, GM-CSF, IFNγ, IL-1α, IL-1β, IL-2, IL-3, IL-4, IL-5, IL-6, IL-9, IL-10, IL-12p40, IL-12p70, IL-13, IL-17A, CXCL-1, MCP-1, MIP-1α, MIP-1β, RANTES, and TNFα were quantified using the Bio-Plex Pro Mouse Cytokine 23-Plex Assay (Biorad) according to the manufacturer’s instructions. Pulmonary TGFβ concentration was quantified by commercial ELISA kit (R&D Systems). For analyses, IL-9 and IL-17A concentrations remained below the limit of detection at all time-points and were excluded.

### Flow cytometry

Lung, spleen, and MLN tissues were harvested, and single cells suspensions were generated by homogenizing tissue through a 100μm nylon filter (Falcon) followed by ACK lysis of red blood cells (Quality biologicals). The total numbers of live cells were determined using a hemocytometer and trypan blue (Invitrogen) exclusion, and cells were resuspended at 1 × 10^6^ cells/ml in RPMI 1640 (Cellgro) supplemented with 10% fetal bovine serum (Fisher Scientific), 1% L-glutamine (Gibco), and 1% penicillin-streptomycin (Gibco). For the enumeration of H1N1-specific CD4^+^ and CD8^+^ T cells, isolated cells were cultured for 5hrs at 37°C in media containing IAV specific peptide (2009ma; CD8: NP_366-374_, or CD4: NP_311-325_, and WSN-OVA_1_; CD8: OVA_257-264_) in the presence of GolgiPlug (BD) and GolgiStop (BD). Following incubation, T cell viability was determined by fixable live/dead far red viability stain (Invitrogen). For all leukocyte populations, Fc receptors were blocked using anti-CD16/32 (BD Biosciences) and panel specific surface markers were stained with the following antibodies: CD4-PerCPCy5.5 (Clone RM1-5; BD), CD8-PerCPCy5.5 (Clone 53–6.7; BD), CD11b-FITC (Clone M1/70; BD), CD11C-APC (Clone HL3; BD), CD25-FITC (Clone 7D4; BD) CD45-PerCPCy5.5 (Clone 30-F11; BD), CD90.1-FITC (Clone OX-7; BD), CD90.2-PE (Clone 53–2.1; BD), CD107a-PE (Clone 1D4B; BD), Ly-6C-PE-Cy7 (Clone AL21; BD), Ly-6G-APC (Clone 1A8; BD), Ly-6G-FITC (Clone 1A8; BD), I-A/I-E (Clone M5/114.15.2), Siglec-F (Clone E50-2440), PE-conjugated tetramer for ma2009 (ASNENVETM; NIH Tetramer Core Facility), and PE-conjugated pentamer for WSN-OVA_1_ (SIINFEKL; Proimmune). Cells were then permeabilized and fixed (BD Cytofix/Cytoperm) prior to intracellular staining with IFNγ-FITC (Clone XMG1.2; BD), IL4-PE (BD), IL17A-PE (BD), and TNFα-PE (Clone MP6-XT22; BD). Intracellular staining with Foxp3 (Clone MF23; BD) was performed following fixation and nuclear permeabilization with the FoxP3/Transcription Factor Staining Buffer Set (eBioscience). Data were acquired using a FACSCailbur flow cytometer (BD) running Cell Quest Pro or an LSRII flow cytometer (BD) running FACSDiva (BD), and analyzed using FlowJo (v.10) software (Tree Star, Inc.). Total live cell counts were determined based on the total live cells counts acquired by trypan blue exclusion staining multiplied by the total live cell percentages for each corresponding gate.

### Lung inflation and histologic analysis

Lungs were inflated with zinc-buffered formalin (Z-Fix, Anatech) under constant pressure (25 cm H_2_), dissected free, and placed in fixative for 48hrs as previously described [80]. Fixed lung tissues were then embedded in paraffin, cut into 5μm sections, and mounted on glass slides. Consecutive tissue sections were subsequently stained with hematoxylin and eosin (H&E) and used to assess eosinophilic inflammation on a 0–3 scale (0, no inflammation; 1, mild inflammation; 2, moderate inflammation; and 3, severe inflammation) for perivascular, peribronchiolar, and alveolar areas. The cumulative eosinophilic score represents the sum of each individual inflammation parameter with scoring performed by a single blinded observer in consultation with a board-certified veterinary pathologist. Representative images were taken at 20x and 200x magnification using a Nikon Eclipse E400 camera.

### *In vivo* eosinophil depletion

Mice were given 75 μg of anti-IL-5 antibody (clone TRFK5; eBioscience) or control IgG1 antibody (clone eBRG1; eBioscience) in 75 μl sterile PBS by IP injection daily from 7 through 12 dpi. Efficacy of pulmonary eosinophil depletion was assessed at 14 dpi by flow cytometry.

### Real time reverse transcription PCR

Pulmonary single cells suspensions were generated by homogenizing lung tissue through a 100μm nylon filter (Falcon) and CD8^+^ T cells were isolated by negative selection (StemCell Technologies). Total RNA was then isolated from purified CD8^+^ T cells using a commercial kit (Invitrogen) per the manufacturer’s instructions and RNA concentration and purity were measured using a NanoDrop (ThermoFisher Scientific). Pre-designed androgen receptor (*Ar)* (Mm.PT.58.12425400) and *Gapdh* (Mm.PT.39a.1) PrimeTime Primers were purchased from Integrated DNA Technologies. Semi-quantitative RT-PCR was performed in 96-well optical reaction plates using SsoFast EvaGreen Supermix (Biorad) on the StepOnePlus RT-PCR system (Applied Biosystems). Gene expression was normalized to *Gapdh* using the ΔCt method.

### Adoptive transfer of CD8^+^ T cells

Splenic tissue was harvested from unprimed gonadectomized male TCR-Ova mice treated with either empty capsules (gdx) or testosterone capsules (gdx + T). CD8^+^ T cells were isolated by tissue homogenization through a 100μm nylon filter (Falcon), followed by negative selection purification (StemCell Technologies). One-hundred thousand purified CD8^+^ T cells were then transferred into gdx and gdx + T treated naive male CD90.1 recipient mice via intravenous inoculation. Recipient mice were infected with WSN-Ova_1_ virus 48 hours post transfer.

### Statistical analysis

Discrete measures were analyzed by one or two-way ANOVA with significant interactions further analyzed using the Tukey method for pairwise multiple comparisons. Repeated measures were analyzed by mixed-effect model with Bonferroni’s post-test for multiple comparisons. Statistical analyses were performed using GraphPad Prism 8.02 software and mean differences were considered significant at *P* < 0.05.

## Supporting information

S1 Table Adult male mice were gonadectomized and implanted with either testosterone (gdx + T) or placebo (gdx) containing capsules, and then inoculated with a sub-lethal dose of ma2009 H1N1 IAV or were mock infected. At 0, 3, 9, or 14 dpi (n = 8-11/treatment/time-point), lung tissue was collected and homogenized, and cell free supernatants were used to quantify pulmonary concentrations of 24 cytokines and chemokines. Data are presented as the mean +/- SEM from 2 independent experiments (n = 8-11/treatment/timepoint) and significant differences between treatment groups for each timepoint are bolded and denoted by asterisks (*P < 0.05).
(DOCX)
