# Supplementary material for: Androgen receptor signaling in the lungs mitigates inflammation and improves the outcome of influenza in mice
Source: PLoS Pathog. 2020 Jul 9;16(7):e1008506. doi: 10.1371/journal.ppat.1008506 (PMC7373319; doi:10.1371/journal.ppat.1008506)
Supplement: S1 Table — At 0, 3, 9, or 14 dpi (n = 8-11/treatment/time-point), lung tissue was collected and homogenized, and cell free supernatants were used to quantify pulmonary concentrations of 24 cytokines and chemokines. Data are presented as the mean +/- SEM from 2 independent experiments (n = 8-11/treatment/timepoint) and significant differences between treatment groups for each timepoint are bolded and denoted by asterisks (*P < 0.05). (DOCX) [file ppat.1008506.s001.docx]

|  | **0dpi** | | **3dpi** | | **9dpi** | | **14dpi** | |
| --- | --- | --- | --- | --- | --- | --- | --- | --- |
| **Cytokine/Chemokine** | **Gdx** | **Gdx + T** | **Gdx** | **Gdx + T** | **Gdx** | **Gdx + T** | **Gdx** | **Gdx + T** |
| IL-1α | 19.2±1.7 | 37.1±5.0 | 25.4±1.9 | 32.2±2.5 | 23.9±2.2 | 33.2±6.0 | 19.0±1.1 | 15.7±1.3 |
| IL-1β | 120.1±6.7 | 151.9±6.9 | 122.3±8.6 | 116.4±5.1 | 253.0±9.3 | 269.2±12.8 | 124.6±7.3 | 145.8±8.2 |
| IL-2 | 15.7±1.4 | 19.0±2.7 | 13.3±1.1 | 11.1±0.9 | 11.2±0.5 | 13.7±0.5 | 6.6±0.1 | 6.3±0.4 |
| IL-3 | 2.2±0.1 | 2.9±0.2 | 3.8±0.4 | 3.6±0.3 | 14.2±1.7 | 12.1±1.3 | 8.5±2.3 | 6.1±0.9 |
| IL-4 | 2.1±0.7 | 2.4±0.6 | 3.4±0.4 | 2.8±0.3 | 6.1±0.6 | 6.6±0.4 | 5.8±0.6 | 4.3±0.4 |
| IL-5 | 6.0±0.7 | 6.4±0.4 | 14.0±1.9 | 8.5±0.9 | 19.1±2.6 | 27.3±2.8 | 20.7±2.5 | 13.9±1.2 |
| IL-6 | 6.7±2.4 | 7.6±3.7 | 114.8±37.1 | 94.2±22.3 | 154.8±35.9 | 219.2±42.2 | 6.7±1.5 | 7.4±1.9 |
| IL-10 | 12.0±0.8 | 13.6±1.0 | 33.4±2.6 | 26.0±1.3 | 103.5±8.2 | 137.5±25.9 | 48.9±2.7 | 45.1±2.7 |
| IL-12 (p40) | 106.2±8.6 | 70.3±4.3 | 501.1±76.3 | 302.8±32.4 | 719.0±53.6 | 599.1±46.1 | 1464.8±190.5 | 1300.9±189.0 |
| IL-12 (p70) | 15.5±1.8 | 14.6±1.1 | 58.4±7.2 | 45.0±3.8 | 83.8±6.8 | 87.8±6.2 | 80.9±7.5 | 63.8±5.6 |
| IL-13 | 57.6±9.2 | 54.9±3.8 | 155.6±11.0 | 133.4±4.0 | 97.7±3.4 | 108.8±3.4 | 135.3±5.3 | 142.0±6.6 |
| Eotaxin | 271.5±22.1 | 286.1±7.9 | 687.0±70.9 | 895.3±72.8 | 267.2±8.8 | 297.0±18.7 | 450.9±142.4 | 423.8±95.3 |
| G-CSF | 5.7±1.7 | 14.7±3.0 | 120.8±22.6 | 209.8±31.3 | 517.5±113.1 | 655.4±70.8 | 62.3±12.0 | 63.1±13.6 |
| GM-CSF | 72.6±6.8 | 69.1±3.7 | 117.7±8.8 | 119.3±7.1 | 61.7±3.6 | 70.4±2.0 | 106.5±7.8 | 96.1±9.1 |
| IFNγ | 10.9±0.9 | 15.6±2.3 | 21.3±2.0 | 19.9±2.7 | 41.1±3.4 | 53.3±10.0 | 12.0±2.1 | 13.2±1.6 |
| CXCL1 | **103.7±20.9** | **683.7±344.5*** | **959.1±161.9** | **1441.5±124.5*** | 527.6±50.7 | 705.5±46.3 | **185.4±12.2** | **816.0±271.2*** |
| CCL2 | 58.4±11.6 | 75.1±8.7 | 2395.5±600.2 | 2150.0±412.5 | 1811.9±155.3 | 2316.8±313.2 | 1599.2±390.2 | 1485.1±233.5 |
| CCL3 | 34.8±2.1 | 31.3±3.9 | 100.3±17.6 | 95.1±11.8 | 1275.3±162.3 | 1894.1±375.7 | 277.8±34.1 | 173.6±27.0 |
| CCL4 | 28.9±1.0 | 33.6±1.9 | 53.2±6.7 | 42.7±3.5 | 455.5±43.7 | 623.4±106.0 | 61.0±11.2 | 45.3±5.4 |
| CCL5 | 304.0±26.0 | 230.9±26.2 | 464.2±61.2 | 375.0±31.0 | 1007.6±176.3 | 1121.6±121.1 | 1222.8±150.9 | 994.8±130.9 |
| TNFα | 81.8±4.8 | 105.0±7.0 | 69.4±4.0 | 62.5±1.8 | 129.0±3.7 | 147.2±6.2 | 62.4±1.7 | 64.5±3.0 |
| TGFβ | 1360.9±222.9 | 1958.8±383.7 | 2317.7±328.6 | 1880.6±127.1 | 1726.8±259.7 | 2604.9±405.2 | 1199.4±128.7 | 1417.4±160.8 |

**Supporting Table 1. Pulmonary cytokine and chemokine concentrations (pg/ml) following IAV infection in gonadectomized mice treated with placebo (Gdx) or testosterone (Gdx + T).**

Data are presented as the mean +/- SEM from 2 independent experiments (n = 8-11/treatment/timepoint) and significant differences between treatment groups for each timepoint are bolded and denoted by asterisks (*P < 0.05).
